# Equivalency of beam scan data collection using a 1D tank and automated couch movements to traditional 3D tank measurements

**DOI:** 10.1002/acm2.12444

**Published:** 2018-09-06

**Authors:** Nels C. Knutson, Matthew C. Schmidt, Matthew D. Belley, Ngoc Nguyen, Michael Price, Sasa Mutic, Erno Sajo, H. Harold Li

**Affiliations:** ^1^ Department of Radiation Oncology Washington University School of Medicine St. Louis MO 63110 USA; ^2^ Medical Physics Program University of Massachusetts Lowell Lowell MA 01852 USA; ^3^ Department of Radiation Oncology Rhode Island Hospital The Alpert Medical School of Brown University Providence RI 02903 USA; ^4^ Education Department Varian Medical Systems Las Vegas NV 89119 USA; ^5^ Department of Physics University of Rhode Island Kingston RI 02881 USA

**Keywords:** 1D Tank, beam scanning using XML, linac commissioning, beam modeling

## Abstract

This work shows the feasibility of collecting linear accelerator beam data using just a 1‐D water tank and automated couch movements with the goal to maximize the cost effectiveness in resource‐limited clinical settings. Two commissioning datasets were acquired: (a) using a standard of practice 3D water tank scanning system (3DS) and (b) using a novel technique to translate a commercial TG‐51 complaint 1D water tank via automated couch movements (1DS). The Extensible Markup Language (XML) was used to dynamically move the linear accelerator couch position (and thus the 1D tank) during radiation delivery for the acquisition of inline, crossline, and diagonal profiles. Both the 1DS and 3DS datasets were used to generate beam models (BM_1_ _DS_ and BM_3_ _DS_) in a commercial treatment planning system (TPS). 98.7% of 1DS measured points had a gamma value (2%/2 mm) < 1 when compared with the 3DS. Static jaw defined field and dynamic MLC field dose distribution comparisons for the TPS beam models BM_1_ _DS_ and BM_3_ _DS_ had 3D gamma values (2%/2 mm) < 1 for all 24,900,000 data points tested and >99.5% pass rate with gamma value (1%/1 mm) < 1. In conclusion, automated couch motions and a 1D scanning tank were used to collect commissioning beam data with accuracy comparable to traditionally acquired data using a 3D scanning system. TPS beam models generated directly from 1DS measured data were clinically equivalent to a model derived from 3DS data.

## 1 INTRODUCTION

While radiotherapy facilities in many countries may have the basic equipment to treat patients with megavoltage radiation, they may be deficient in expensive QA equipment and/or the expertise afforded by trained professionals to perform complex quality assurance procedures, and this disparity in hardware and professional resources is concerning on the global scale.^1^ This is particularly apparent when looking at the discrepancy in the access to advanced treatment modalities for low‐ and middle‐income countries vs high‐income countries.^2^ Efforts to solve this problem continue as the National Institute of Health has recently announced funding opportunities for the development of cancer‐relevant technologies for low‐ and middle‐income countries (RFA‐CA‐15‐024).^3^ There are many challenges to overcome in radiation oncology; acquisition of commissioning beam data is a prime example.

The World Health Organization estimated that approximately 750 of 3125 (24%) reported adverse advents in radiation oncology stemmed from the commissioning stage.^4^ Beam data acquisition is an important step in the commissioning process, as it is the foundation for subsequent beam modeling. Errors made during beam data acquisition and modeling are particularly hazardous, since these errors will be systematic and propagate to impact every patient treated on a given machine. Therefore, it is crucial this process be accurate and error free. The beam data acquisition process involves the use of sophisticated scanning software to position the detector and take readings; however, this is often labor intensive. Beam scanning systems are not integrated with treatment systems as changes in the scanning software do not automatically translate to changes in the machine parameters (e.g., jaw settings or energy selection) and thus can be error prone (AAPM TG‐106).^5^ Furthermore, beam modeling becomes more critical as the complexity of treatment increases (e.g., SBRT & IMRT).^6^ Currently, guidelines exist for ensuring best practices during the beam scanning process,^5^ treatment planning system commissioning process,^6^ and in the continued quality assurance of treatment planning systems.^7^ The task groups underscore the importance of using precise and accurate equipment that, on the other hand, can come at a high financial cost. Furthermore, despite the presence of these guidelines, there is still substantial variability in the quality and accuracy of commissioning in the United States as seen by third party audits of institutions.^8^, ^9^, ^10^, ^11^ One possible cause could be a shortage for personnel proficient in these procedures to provide services.^12^

This work presents a novel method to lower the financial and equipment barriers needed to acquire a full dosimetric commissioning dataset by presenting a departure from traditional non‐integrated 3D scanning systems (3DS), and by transitioning to the synergistic and efficient use of a compact 1D water tank and automated translation of the linear accelerator couch system (1DS) via the extensible markup language (XML). The logistical characteristics of the 1DS and 3DS systems per the manufacturer's technical data sheet highlight the differences between the two systems. The 3D scanning system tank (diameter = 87.5 cm, height = 67.3 cm) requires a stand (123 × 113 × 58.4 cm^3^) and may use an optional reservoir (114.2 × 65 × 90 cm^2^) when tissue maximum ratio (TMR) measurements are needed. In aggregate, the onerous amount of equipment (1.88 m^3^ and 382.7 kg) poses a high cost for shipping and takes up valuable space for onsite storage. The purchase price of a 3D system is approximately $100,000 without considering recurring maintenance and storage costs. In comparison, the purposed 1D system with automated couch motions improves cost (≈$10,000), size and form factor (37.6 × 40.6 × 36.8 cm^3^), and weight (10 kg empty and 64 kg full). Not including storage and maintenance, the 1D tank leads to a savings of $90,000, 1.857 m^3^, and 318.7 kg for the system, representing a major improvement that could be particularly impactful in developing countries.

## 2 MATERIALS AND METHODS

### 2.A Data collection

Two commissioning datasets were acquired: (a) using a standard 3D water tank scanning systems (3DS) and (b) using a 1D tank with automated couch movements (1DS) for a 6MV beam from a commercial linear accelerator (TrueBeam, Varian Medical Systems, Palo Alto, CA). Each dataset was collected using field and reference 0.13 cc (3.0 mm radius) scanning ionization chambers (CC13, IBA Dosimetry, Schwarzenbruck Germany) accounting for the effective point of measurement of the chamber. All 1DS and 3DS scans were completed continuously at 2.5 mm/s with data spacing of 1.25 mm. In all datasets, central axis depth profiles and lateral profiles were collected for 3 × 3, 4 × 4, 6 × 6, 8 × 8, 10 × 10, 20 × 20, 30 × 30, and 40 × 40 cm^2^ field sizes. The lateral profiles consisted of inline and crossline profiles. 45 degree diagonal profiles from (−X, −Y) to (+X, +Y) were taken for the 40 × 40 cm^2^ field size. All profiles were taken at depths of 1.5, 5, 10, 20, and 30 cm. In each profile scans went 5 cm past the geometric width of the field providing 5 cm of over‐scan on each side of the profile. The depth profiles were collected from 30 cm depth to the water surface to minimize the disturbance of the water surface.

For the 1DS system, a TG‐51 compliant 1D water tank was placed on the treatment couch on top of a 40 × 40 × 5 cm^3^ slab of water equivalent plastic to provide additional backscatter for 30 cm depth measurements. Using a mechanical front pointer, the water surface was set to 100 cm source‐to‐surface distance (SSD). The field scanning ionization chamber was aligned to the crosshair of the gantry and checked to be level and plumb using the gantry and a spirit level. The reference detector was fixed and aligned in the corner of the radiation field not obscuring the field chamber independent of the couch position. TrueBeam Developer Mode (Varian Medical Systems, Palo Alto, CA) was used to control the linear accelerator using the Extensible Markup Language (XML). Developer Mode is the commercially available solution that allows customers easy access to deliver XML based plans that can dynamically move the couch during delivery with an accuracy of approximately 0.1 mm due to translational errors or vertical sag.^13^ XML files were used to dynamically move the couch during radiation delivery across the beam for inline, crossline, and diagonal profile measurements. Profiles were collected by moving the couch as a function of MU delivered. To allow for the greatest scan length the couch was moved in the longitudinal direction at different couch angles to complete inline, crossline, and diagonal scans. Scans were completed at 600 MU/min with a couch speed of 2.5 mm/s with the long axis of the chamber oriented perpendicular to the couch motion. 2.5 mm/s allowed for a balance of scan speed and lack of water surface motion. The depth of the chamber was controlled by the 1D tank software while charge readings were recorded by a data logging electrometer every 500 ms. This data was then saved for analysis via software developed in house. The couch angle and tank orientations were adjusted for inline, crossline, and diagonal scans to scan along the short axis of the chamber and ensure proper alignment of the chamber to isocenter, while mitigating dependence on the couch walk out.

Each profile collected via the 1DS was then compared and plotted to the paired profile from the 3DS dataset using a custom 1D gamma analysis code^14^ using dose difference and distance to agreement criteria to calculate a set of gamma values for each set of profiles compared. The central axis depth profiles were normalized to the maximum dose and 1% dose difference and 1 mm distance to agreement gamma criteria were used. Lateral profiles were normalized to the central axis of each profile and 2%/2 mm gamma criteria was used to analyze all profiles. No smoothing was used on either data set.

### 2.B Beam modeling and comparison

Upon collection of all required data with the 1DS and 3DS systems, all data were formatted for import into the treatment planning system (Eclipse 13.7, Varian Medical Systems, Palo Alto, CA). For each dataset a 6 MV beam model was created using the Analytical Anisotropic Algorithm (AAA).^15^, ^16^, ^17^ The resultant beam models (BM_1DS_ created from the 1DS data and BM_3DS_ from the 3DS data) were then used to calculate a number of static open fields in the treatment planning system on a 70 × 70 × 70 cm^3^ virtual water phantom. These fields included: 2 × 2, 3 × 30, 4 × 16, 5 × 5, 5 × 10, 7 × 7, 10 × 5, 10 × 10, 15 × 15, 16 × 4, 25 × 25, 30 × 3, 35 × 35 cm^2^ field sizes. Two dynamic MLC fields were calculated, the dynamic chair^18^ and pyramid fields,^19^ in addition to open static fields. These dynamic MLC fields are representative of dynamic MLC fields used to treat patients. The resultant dose distributions from the two beam models (BM_1DS_ and BM_3DS_) were compared using an extension of the 1D gamma analysis,^14^ a 3D gamma metric tool^20^, ^21^ that was scripted within the treatment planning system leveraging the Eclipse Scripting Application Programming Interface (ESAPI). In each case, 3D gamma value distributions (1 × 1 × 5 mm^3^ point spacing) were calculated. To reduce computation time a dose threshold of 5% of the maximum dose was used. The 2D planes from the 3D gamma distributions for axial, coronal, and sagittal planes were extracted, plotted, and reviewed.

## 3 RESULTS

### 3.A Beam data collection

**Figure 1:**
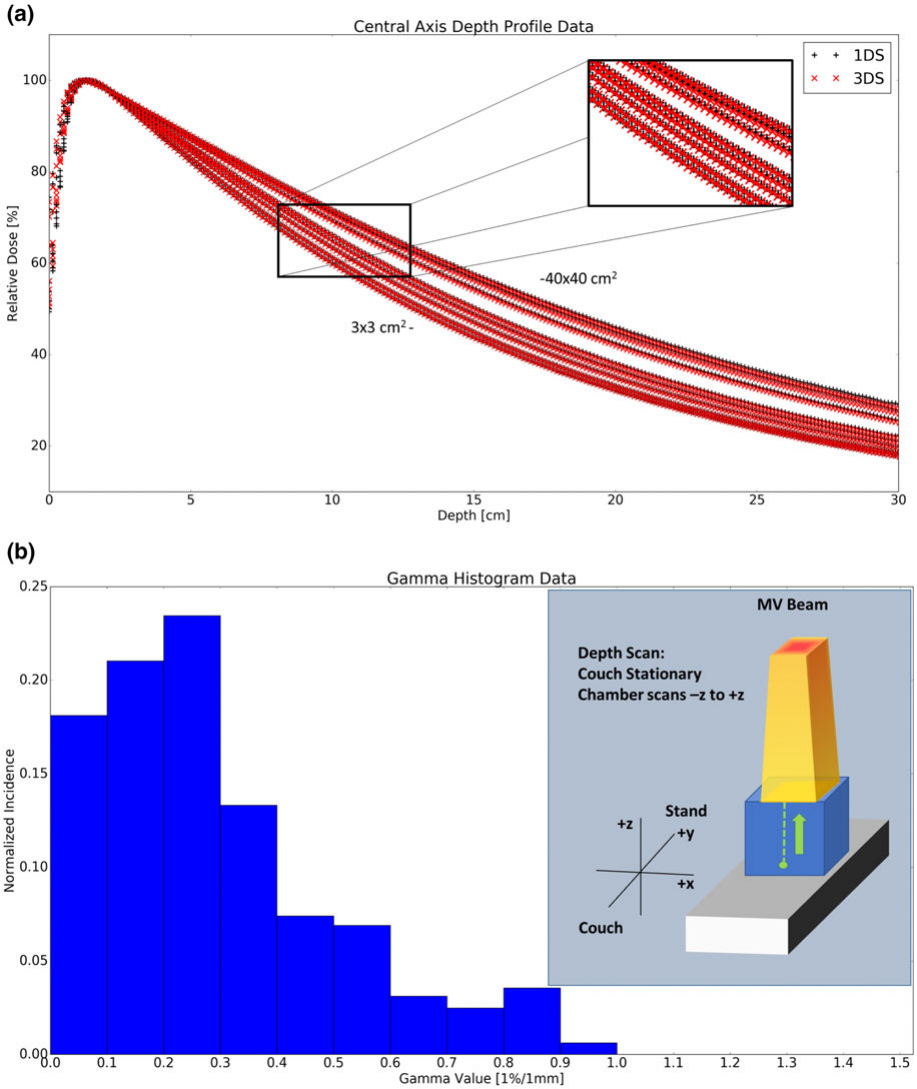
6 MV Central axis depth scan data as a function of square field sizes (1a) 3 × 3, 4 × 4, 6 × 6, 8 × 8, 10 × 10, 20 × 20, 30 × 30, & 40 × 40 cm^2^. (1b) The corresponding histogram of 1D gamma values for the curves in Fig. 1(a).

Table 1 compares the 1DS vs 3DS central axis depth scan data collected. Both systems agreed well with >99.9% of data points yielding a gamma value <1 with a 1%/1 mm gamma criteria. The maximum gamma value was 1.523, however this was located in the buildup region near the water surface. At depths deeper than 0.5 cm all points were well within the 1%/1 mm gamma criteria with a mean gamma value of 0.254 across all field sizes. The depth profile data as a function of field size [Fig. 1(a)] and histogram of the gamma values from these profiles [Fig. 1(b)] highlight this agreement.

**Table 1:** 1D gamma comparison of 1DS PDD data to 3DS central axis depth profile data (Γ: 1%/1 mm)

Field size (cm^2^)	1DS vs 3DS		
	% points Γ < 1	Mean Γ	Max Γ
3 × 3	100	0.101	0.916
4 × 4	100	0.174	0.916
6 × 6	100	0.195	0.822
8 × 8	100	0.259	0.815
10 × 10	100	0.266	0.810
20 × 20	100	0.395	0.704
30 × 30	99.9	0.539	1.523
40 × 40	100	0.386	0.992
All field sizes	99.9	0.290	1.523

**Figure 2:**
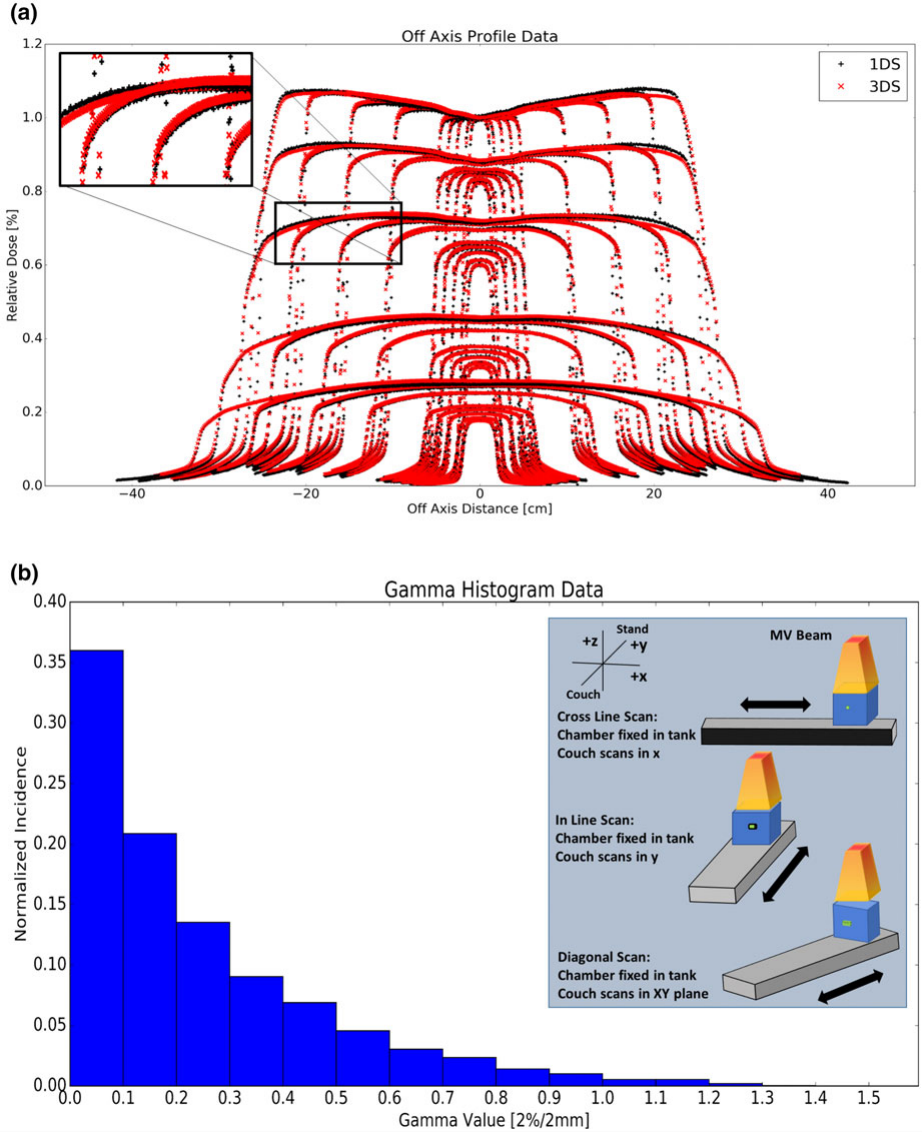
6 MV lateral profile data as a function of square field sizes (2a) 3 × 3, 4 × 4, 6 × 6, 8 × 8, 10 × 10, 20 × 20, 30 × 30, & 40 × 40 cm^2^. (2b) The corresponding histogram for all lateral profile data in 2(a).

All measured profiles were compared at 2%/2 mm and these data are summarized in Table 2. Over 98.7% of all data points yielded gamma values <1. The mean gamma value across all profiles was 0.241. Figure 2(a) shows the off axis profiles for the various field sizes and depths. Note for plotting, all profiles were normalized to the central axis of a 10 × 10 cm^2^ at 1.5 cm depth; however, for the gamma analysis each profile was normalized to its own central axis. A histogram of the gamma values for these profiles is plotted in Fig. 2(b).

**Table 2:** 1D gamma comparison (2%/2 mm) of 1DS lateral profile data to 3DS lateral profile data

Field size (cm^2^)	(Γ: 2%/2 mm)		
	% points Γ < 1	Mean Γ	Max Γ
3 × 3	100	0.102	0.668
4 × 4	100	0.084	0.617
6 × 6	100	0.092	0.655
8 × 8	100	0.107	0.660
10 × 10	100	0.125	0.715
20 × 20	99.9	0.256	1.040
30 × 30	96.6	0.312	1.284
40 × 40^a^	98.0	0.329	1.593
All field sizes	98.7	0.241	1.593

a Includes diagonal profiles.

### 3.B Beam modeling

**Figure 3:**
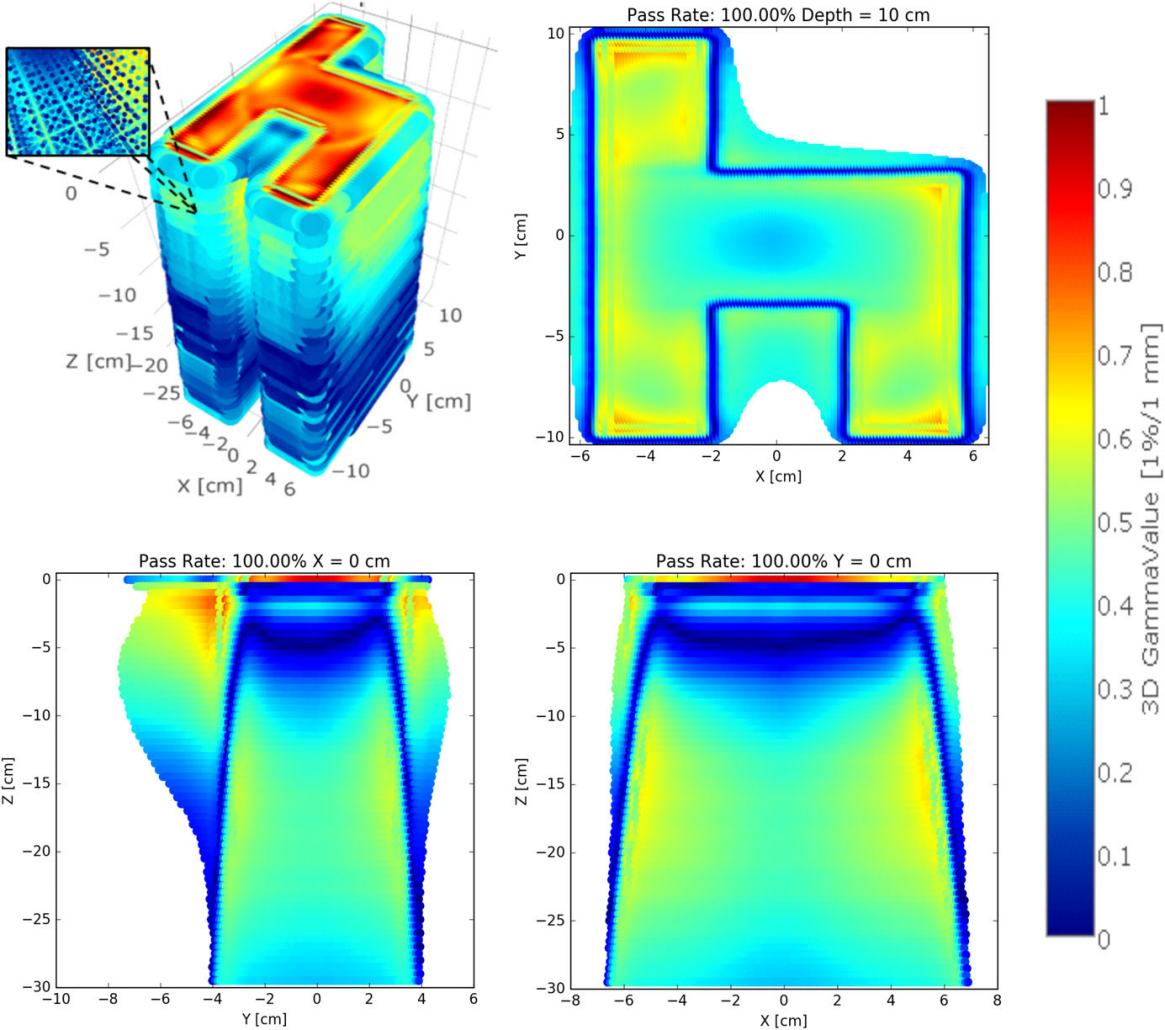
3D gamma value distribution and corresponding axial, coronal, and sagittal planes for the Chair test pattern resulting from BM_1_ _DS_ and BM_3_ _DS_ computed dose. The inlay figure shows a zoomed in view of the 3D gamma value distribution.

**Figure 4:**
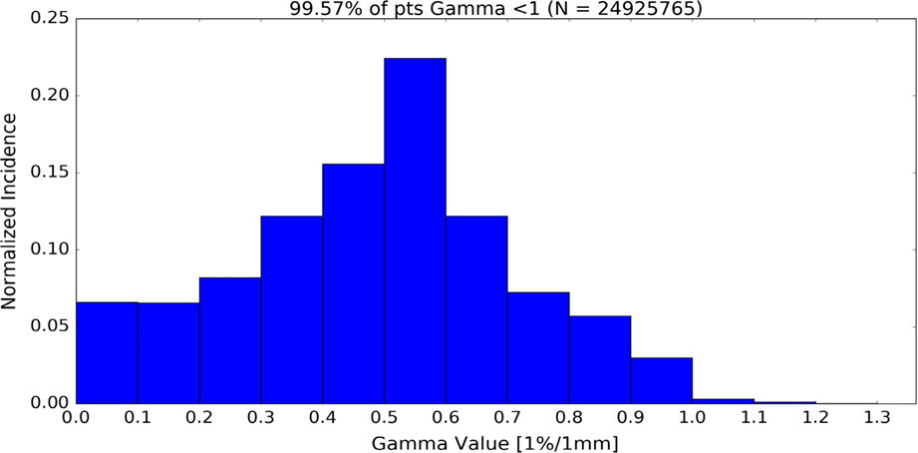
Histogram data of gamma values (1%/1 mm) across all field sizes compared for computed dose of beam models BM_1_ _DS_ and BM_3_ _DS_.

To quantify the differences in the two beam models created (BM_1DS_ and BM_3DS_), beams of various field sizes were calculated on a water phantom in the treatment planning system and compared using a 3D gamma metric for each field size. The results are summarized in Table 3. The 3D gamma value distributions (1 × 1 × 5 mm^3^ point spacing) for all fields sizes led to greater than 24,900,000 data points being compared in total with excellent agreement. The dose distribution comparison for the TPS beam models BM_1DS_ and BM_3DS_ had 3D gamma value (2%/2 mm) < 1 for all points analyzed; and >99.5% pass rate with gamma value (1%/1 mm) < 1. An example analysis showing the 3D gamma value distribution, axial, coronal, and sagittal planes for the dynamic chair field is presented in Fig. 3. Histogram data of the gamma values were collected and are presented in Fig. 4.

**Table 3:** 3D gamma comparison (1%/1 mm 5% threshold) of dose distributions calculated from the BM_1DS_ and BM_3DS_ beam models

Field size X × Y (cm^2^)	(Γ: 1%/1 mm)			
	# of data points	% points Γ < 1	Mean Γ	Max Γ
2 × 2	3.95 × 10^4^	100.00	0.241	0.706
3 × 30	8.25 × 10^5^	99.99	0.333	1.363
4 × 16	5.77 × 10^5^	99.99	0.318	1.145
5 × 5	2.27 × 10^5^	100.00	0.299	0.908
5 × 10	4.47 × 10^5^	100.00	0.330	0.889
7 × 7	4.36 × 10^5^	100.00	0.322	0.917
10 × 5	4.47 × 10^5^	100.00	0.319	0.787
10 × 10	8.77 × 10^5^	99.99	0.354	1.044
15 × 15	1.95 × 10^6^	99.99	0.376	1.123
16 × 4	5.76 × 10^5^	100.00	0.327	0.937
25 × 25	5.36 × 10^6^	99.20	0.522	1.215
30 × 3	8.17 × 10^5^	99.99	0.335	1.109
35 × 35	1.05 × 10^7^	99.41	0.570	1.356
Dynamic chair (12 × 20)^a^	1.20 × 10^6^	99.97	0.401	1.171
Pyramid field (12 × 25)^a^	6.64 × 10^5^	99.66	0.482	1.254
All fields	2.49 × 10^7^	99.57	0.483	1.363

a Dynamic MLC fields.

## 4 DISCUSSION

The 1DS measured data show excellent agreement with the data measured with the 3DS system. Outside of the buildup region, all central axis data were within 1%/1 mm gamma criteria. Off axis, 98.5% of points were within 2%/2 mm with the only discrepancies >2% but <3% being seen in the largest field sizes 30 × 30 and 40 × 40 cm^2^ in the low‐dose tails beyond the 80%‐20% penumbra. These discrepancies could be due to the difference in scatter geometry between the two measurement setups for large fields beyond the field edge in particular at deep depths. Agreement at the 1%/1 mm for smaller fields (~10 × 10 cm^2^) and increased discrepancies for larger fields (40 × 40 cm^2^) in low‐dose region have been described in other works as well.^22^, ^23^, ^24^, ^25^ 40 × 40 cm^2^ fields are rarely used in a clinical treatment planning process. These large field sizes are generally used for total body irradiation and most commonly a hand calculation is used instead of a volumetric dose calculation. Furthermore, the treatment geometry for these treatments does not reflect the measurement geometry used during the standard commissioning process.

In addition to the measurement data, the resultant beam model in the treatment planning system is of most importance as this is what is used to calculate dose to the patient and derive the monitor units that the patient will ultimately be treated with. The resultant dose distributions from the two different scanning systems (1DS & 3DS) show excellent agreement, yielding beam models (BM_1DS_ and BM_3DS_) that calculate dose with greater than 99.5% of data points with a gamma value (1%/1 mm) < 1. Similar to the measured data, the only discrepancies greater than 1% but less than 2% were seen either within 5 mm of the phantom surface in the buildup region and/or in large square field sizes, e.g., 35 × 35 cm^2^. In more clinically realistic field sizes where at least one axis is less than 20 cm wide, including dynamic MLC delivery fields, this discrepancy is not present. Greater than 99.9% of points had a gamma value (1%/1 mm) less than 1, thereby yielding two clinically equivalent beam models in our treatment planning system. Although our results are promising, different users and/or treatment planning systems may yield different results and further investigation is warranted.

A 40 × 40 cm^2^ diagonal scan at 100 cm SSD at 30 cm depth with 5 cm of over scan can be acquired in a single 83.5 cm long continuous scan with our 1D tank method; a feat that is not possible with current 3D systems. With the 1DS system the ion chamber maintains a constant amount of scatter material (approximately 15 cm of water) on each side of the chamber in contrast to the 3DS system which translates the chamber near the edge of the tank during a profile scan, thus potentially reducing the scatter around the chamber as compared to central axis for large fields. The 1DS system can reduce the time and complexity in accurately collecting large field scans.

In recent years, several ring gantry geometry machines have been developed, e.g., the Halcyon (Varian Medical Systems, Palo Alto, CA), the MRIdian (ViewRay, Oakwood Village, OH), the Unity (Elekta, Stockholm, Sweden), and TomoTherapy (Accuray, Sunnyvale, CA). Ring gantry geometry does not lend itself to the use of large 3D tanks for commissioning. A smaller and more compact 1D system described in this work may allow for the collection of commissioning beam data or at the very least, a more straight forward independent validation of beam data process in water in this geometry.

Developer Mode was used for this work due to the simplicity of the XML interface. However, it is worth noting that this technique can be implemented and utilized even without Developer Mode. Previous work has shown the measurement of TMRs by manual control of the couch motion^26^. Existing delivery platforms such as Halcyon, TrueBeam, and VitalBeam incorporate a hardware key that Varian service may use to enable delivery of automated XML plans without purchasing Developer Mode. Future development of automated and integrated systems that connect the linear accelerator and the dosimetry equipment could potentially lead to a more reproducible measurement system, which could potentially reduce the inconsistencies currently seen in commissioning due to user error via automation, while increasing access for low resource areas to the high quality commissioning of the complex treatment systems in radiation oncology today. The current 1DS method is comparable in speed to traditional 3DS methods, however, automation could vastly improve this. Future work will include developing tools for automating this process, developing the necessary and extensive quality assurance procedures required to ensure this process remains accurate and stable, automating this quality assurance, and studying if there are any long term effects on the treatment couch, which have not been seen thus far in this preliminary work.

Many of the above and previously discussed limitations, complexities, and impracticalities of the commissioning process stem from the use of a large 3D tank. This has led several researchers to a search for alternative ways to commission, validate, and perform continuous QA on linear accelerators without the use of a 3D tank, including the use of 2D tanks, electronic portal imaging devices, and ion chamber arrays.^27^, ^28^, ^29^, ^30^ This work shows a novel water based dosimetry method for photon beam commissioning, validation, and periodic quality assurance of linear accelerators with improvements in cost, size, and technical burden of the 1DS vs 3DS. Using a TG‐51 compliant tank, readily available in most departments, and the appropriate detectors of choice, one can now commission, validate, and perform annual/periodic QA with a device that fits in the trunk of a car as compared to 3D scanning systems that need to be transported via moving truck.

Even though there appears to be a logistical difference between 1DS and 3DS, many of the short comings of 3D tanks have been addressed in 2D tanks which are substantially cheaper and hence do find wide clinical acceptance worldwide. Even though the 1DS appears to be promising, there is substantial initial development time and QA cost as no commercial system is yet available. This is important as 2D/3D scanners need FDA‐510k clearance before they can be sold. The latter assures the users about the quality of the system. Barring some electronic or motor drive assembly space, nearly the entire volume of the 2D/3D tank is available for data acquisition. This information is available as a specification of the tank by the company. With the 1DS, the free 3D space around couch restricts its range and can vary from linear accelerator type to another. The motion mechanism of a 2D/3D scanner is usually used very infrequently in a clinic and usually gets minimal wear and tear and hence results may be more trustworthy. The 1DS, on the other hand, relies on couch motion accuracy which is subjected to continuous and torturous use every day implying more wear and tear. Hence, every time the 1D tank is used, extensive QA on the couch needs to be carried out. Currently, the system could only be used if the linear accelerator is relatively new supporting XML language for its couch control. In a department which has a mix of different linear accelerator types, it might be more cost effective to have a 2D/3D tank based scanning system which can be used with any of them.

[Correction added on September 14 2018, after first online publication: Under Discussion section "The latter assures the users about the quality of the system." sentence was modified.]

## 5 CONCLUSION

Using a 1D tank and automated couch motions, a full 6 MV commissioning dataset was collected and produced a beam model clinically equivalent to traditional 3D tank based methods. This method could provide a valuable alternative option for commissioning a linac in developing and resource‐limited countries, or for systems where the 3D tank is not feasible.

## CONFLICT OF INTERESTS

The authors declare no conflict of interest.
